# Resveratrol improves cardiac function and left ventricular fibrosis after myocardial infarction in rats by inhibiting NLRP3 inflammasome activity and the TGF-*β*1/SMAD2 signaling pathway

**DOI:** 10.7717/peerj.11501

**Published:** 2021-05-28

**Authors:** Jinjin Jiang, Xiuping Gu, Huifeng Wang, Shibin Ding

**Affiliations:** 1 Jiangsu Vocational College of Medicine, Yancheng, Jiangsu, China; 2 Department of Cardiology, General Hospital of TISCO, Taiyuan, Shanxi, China

**Keywords:** Resveratrol, Cardiac function, Left ventricular fibrosis, NLRP3 inflammasome, Acute myocardial infarction

## Abstract

**Background:**

Several studies have shown that resveratrol (RES), a naturally occurring polyphenol found in many plants, is beneficial for preventing cardiovascular diseases. However, the mechanism underlying the RES-mediated protection against myocardial infarction has not yet been revealed entirely. In this study, we investigated the protective effects of RES on cardiac function in a rat model of acute myocardial infarction (AMI) and the related underlying mechanisms.

**Methods:**

Male Sprague-Dawley rats were randomly divided into four groups: Sham (sham operation), Sham-RES, AMI (AMI induction), and AMI-RES. The rat AMI model was established by the permanent ligation of left anterior descending coronary artery method. The rats in the RES-treated groups were gavaged with RES (50 mg/kg/day) daily for 45 days after the Sham operation or AMI induction; rats in the Sham and AMI groups were gavaged with deionized water. Cardiac function was evaluated by echocardiography. Atrial interstitial fibrosis was assessed by hematoxylin-eosin or Masson’s trichrome staining. Real-time PCR and western blotting analyses were performed to examine the levels of signaling pathway components.

**Results:**

RES supplementation decreased the inflammatory cytokine levels, improved the cardiac function, and ameliorated atrial interstitial fibrosis in the rats with AMI. Furthermore, RES supplementation inhibited NLRP3 inflammasome activity, decreased the TGF-*β*1 production, and downregulated the p-SMAD2/SMAD2 expression in the heart.

**Conclusion:**

RES shows notable cardioprotective effects in a rat model of AMI; the possible mechanisms underlying these effects may involve the improvement of cardiac function and atrial interstitial fibrosis via the RES-mediated suppression of NLRP3 inflammasome activity and inhibition of the TGF-*β*1/SMAD2 signaling pathway in the heart.

## Introduction

The World Health Organization has predicted that 23.3 million people will have died annually from coronary artery disease (CAD) by 2030 (Akila & Vennila, 2016). Acute myocardial infarction (AMI) is one of the main causes of the mortality associated with cardiovascular diseases (CVDs) worldwide. AMI, characterized by cardiomyocyte necrosis and acute loss of myocardial tissue, is a common complication of CAD. To preserve cardiac function and minimize the diastolic and systolic wall stress caused by AMI, the structural and biomechanical response in the heart leads to several changes including collagen deposition with scar formation, fibrosis, hypertrophy, and modifications in the architecture of the left ventricle. All these changes are called “ventricular remodeling”, which affects ventricle function and the patients’ prognoses after AMI (Eaton et al., 1979; McKay et al., 1986), ultimately increasing the risk of heart failure (Anderson & Morrow, 2017). Therefore, reversing ventricular remodeling and improving the cardiac function are critical for ensuring a favorable prognosis in patients with AMI.

Resveratrol (RES), a naturally occurring polyphenolic phytoalexin found in many plants (such as grapes, cranberries, and polygonum bat), has attracted extensive attention for its ability to prevent CVDs. Epidemiologic studies have reported that the consumption of red wine is closely associated with the decreasing incidence of CVDs (Di Castelnuovo et al., 2002; Rendig et al., 2001), and RES has been believed to be responsible for these beneficial effects (Balestrieri et al., 2008; Wu & Hsieh, 2011). In the past two decades, many preclinical and some pilot clinical studies have reported that RES supplementation shows cardioprotective effects in humans (Tome-Carneiro et al., 2013; Zordoky, Robertson & Dyck, 2015). RES plays a protective role against CVDs, owing to its protective action on the vascular walls against platelet oxidation, inflammation, oxidation, and thrombus formation (Delmas, Jannin & Latruffe, 2005). Previous studies have demonstrated that RES supplementation prevents or reverses cardiac dysfunction and remodeling in various animal models of CVDs (Ahmet et al., 2017; Sung et al., 2015; Thandapilly et al., 2010). Some studies have reported that supplementation with low doses of RES (0.1, 5.0, and 17 mg/kg/day) shows no cardioprotective effects in rodents with AMI (Kanamori et al., 2013; Lin et al., 2008). Interestingly, intervention with moderate-high doses of RES (50 mg/kg/day) has been shown to partially reverse left ventricular dilation and significantly improve the cardiac function in mice with AMI via the enhancement of the autophagy-activating AMP-kinase pathway (Kanamori et al., 2013). RES exerts cardioprotective effects due to its ability to stimulate signaling molecules in the cardiovascular system, including AMP-activated protein kinase (AMPK), nuclear factor kappa B (NF- *κ*B), endothelial nitric oxide synthase (eNOS), nuclear factor (erythroid-derived 2)-like 2 (Nrf2), Akt, peroxisome proliferator-activated receptor- *α* coactivator 1 (PGC-1 *α*), silent information regulator 2/sirtuin-1 (SIRT-1), and endogenous anti-oxidant enzymes (Bonnefont-Rousselot, 2016; Dolinsky & Dyck, 2011). However, the cardioprotective mechanism of RES whereby it is associated with the nucleotide-binding domain and leucine-rich repeat protein 3 (NLRP3) inflammasome signaling pathway in an animal model of AMI remains unclear.

A previous study has confirmed that the inflammatory process caused by myocardial ischemia-reperfusion injury can be alleviated by ultramicronized palmitoylethanolamide treatment (Di Paola et al., 2016). A new compound containing palmitoylethanolamide and baicalein treatment also decreases myocardial tissue injury, proinflammatory cytokines (TNF-*α*, IL-1*β*) production in a model of myocardial ischemia-reperfusion (D’Amico et al., 2019). Furthermore, pistachios reduces cardiac tissue injury during acute ischemia-reperfusion through modulating inflammation process in diabetic streptozotocin-induced hyperglycaemic rats (Di Paola et al., 2018). Therefore, the inflammatory response is one of the most key processes in the myocardial ischemia-reperfusion injury. The NLRP3 inflammasome, an important part of the innate immune system, has been demonstrated to recognize and respond to danger signals and cause a series of inflammatory responses in many diseases (such as atherosclerosis and pulmonary interstitial fibrosis) (Chen & Nunez, 2010). Recent evidence has supported that NLRP3 inflammasome activation contributes to inflammatory responses, adverse cardiac remodeling, cardiac dysfunction, and heart failure in animal models of AMI (Toldo & Abbate, 2018). Nevertheless, the role of the NLRP3 inflammasome during the recovery period following AMI is not yet well understood. Previous studies on rodents have suggested that RES treatment can inhibit the NLRP3 inflammasome activity, thereby ameliorating hepatic meta-flammation and attenuating early brain injury and cerebral ischemia/reperfusion injury (He et al., 2017; Yang & Lim, 2014; Zhang et al., 2017). In addition, previous data have also demonstrated that RES shows protective effects against lung fibrosis by inhibiting NLRP3 inflammasome activation in mice with lipopolysaccharide-induced acute lung injury (Yang & Lim, 2014). These findings suggest that the ability of RES to inhibit NLRP3 inflammasome activation may serve as a potential therapeutic strategy against the progression of post-infarction ventricular remodeling. However, whether RES intervention can alleviate AMI-induced left ventricular fibrosis and cardiac dysfunction by inhibiting NLRP3 inflammasome activation remains unclear. In this study, we aimed to determine whether moderate-high doses of RES prevent or reverse the loss of cardiac function and ameliorate left ventricular fibrosis in a rat model of AMI, and further explored the role of the NLRP3 inflammasome signaling pathway during the improvement of cardiac function and reversal of structural remolding in the hearts of RES-treated rats with AMI.

## Material and Methods

### Reagents and antibodies

RES (3,4,5-trihydroxy-trans-stilbene) was purchased from Sigma-Aldrich (St. Louis, MO, USA). The rabbit anti-NLRP3 (cat.DF7438), rabbit anti-caspase-1 p10 (cat.AF4022), and anti-TGF-*β*1 primary antibodies (cat.AF1027), and the goat anti-rabbit IgG (H+L)-HRP (cat.S0001) and goat anti-mouse IgG (H+L)-HRP (cat.S0002) secondary antibodies were purchased from Affinity Biosciences, Inc. (Cincinnati, OH, USA). The primary rabbit anti-apoptosis-associated speck-like protein containing a CARD (ASC) antibody was purchased from Santa Cruz Biotechnologies (cat. sc271054; Santa Cruz, CA, USA). Rabbit anti-SMAD-2 (cat.5339), rabbit anti-p-SMAD-2 (Ser465/467) (cat.18338) and mouse anti-GAPDH (cat.8884) antibodies were obtained from Cell Signaling Technology (Billerica, MA, USA).

### Animal care and study design

Male Sprague-Dawley rats (weighing 210–240 g) were purchased from the Vital River Laboratory Animal Technology Co., Ltd. (Beijing, China). The rats were maintained individually at controlled conditions of temperature (20 °C–22 °C) and humidity, with a 12-h light/12-h dark cycle. All rats had free access to food and water during the study. The animal care procedures in the study were approved by the institutional animal research ethics committee of the General Hospital of TISCO (ethics reference number: TISCO2019037). All animals were treated humanely. The study has established the criteria for euthanasia before the planned trial ends, and the rats that died while under anesthesia were disinfected and buried.

After 7 days of acclimation, a total of 40 male rats were randomly divided into 4 groups (*n* = 10 in each group): (1) The Sham group: underwent sham operation; (2) The Sham-RES group: underwent sham operation and received RES (50 mg/kg/day) supplementation; (3) The AMI group: underwent AMI induction; (4) The AMI-RES group: underwent AMI induction and received RES (50 mg/kg/day) supplementation. Rats in the Sham-RES and AMI-RES groups received RES (dissolved in 0.5 mL of deionized water) by oral gavages daily for 45 days after the sham operation or AMI induction. Meanwhile, the rats in the Sham and AMI groups were gavaged with 0.5 mL of deionized water. In the past decades, RES has been studied as complementary and alternative medicine (CAM) for the therapy of metabolic diseases and cancer (Berretta et al., 2017). Thus, the RES translation dose used in human should be calculated. According to a previous literature (Reagan-Shaw, Nihal & Ahmad, 2008), the dose translation of RES used in our study from rat to adult human dose was about 8.11 mg/kg.

The rat AMI model was produced as described in a previous study (Qiu et al., 2018b). First, the rats were anesthetized via the intraperitoneal injection of 10% chloraldurate solution. Then, they were placed on a ventilator; they underwent left lateral thoracotomy, followed by pericardiectomy to expose their hearts. Next, their left anterior descending coronary arteries were ligated at a distance of two mm below the left atrial appendage and sewn using 5-0 silk sutures to establish the AMI model. Rats in the Sham group underwent thoracotomy and pericardiectomy, but the sutures were attached under their left anterior descending coronary arteries without ligation. At 24 h after the operation, coronary artery occlusion of the rats was confirmed using echocardiographic images. Eighty percent of the rats survived the AMI induction procedure. On day 45 after the operation, the other rats in each group were sacrificed via intravenous pentobarbital injection (20 mg/kg) and exsanguination after the assessment of their cardiac function. After the rats were sacrificed, their hearts were collected, weighed, and frozen in liquid nitrogen immediately for further studies. The remaining hearts were fixed using 4% formaldehyde solution for histological analysis.

### Determination of the serum levels of natriuretic peptide (BNP), interleukin-6 (IL-6), C-reactive protein (CRP), and tumor necrosis factor-alpha (TNF-*α*)

Blood samples were collected from the rats that had been fasted for 12 h. The sera from the blood samples were extracted by centrifugation (1,000 g for 10 min at 4 °C) and used for the quantification of cytokines (including BNP, IL-6, CRP, and TNF-*α*) using the respective commercial ELISA kits (BIOTECH CO., Wuhan, China), according to the manufacturer’s instructions.

### Cardiac function analysis using echocardiography

Forty-five days post the induction of AMI, echocardiographic images of the rats were recorded using a Vivid 7 instrument (GE Co., Inc., Schenectady, New York, USA) equipped with a 12-MHz transducer. Two-dimensional gray-scale echo images and left ventricular M-mode images were obtained during each echocardiographic examination. The left ventricular end-systolic diameter (LVESD), left ventricular end-diastolic diameter (LVEDD), fractional shortening (FS), and ejection fraction (EF) were measured automatically in the M mode by averaging the data from 3 consecutive cardiac cycles. The image acquisition and offline measurements were performed by two technicians who were blinded to the treatments.

### Atrial fibrosis assay

Formaldehyde (4%)-fixed and paraffin-embedded heart sections (5-µm-thick sections) were subjected to hematoxylin-eosin (H&E) or Masson’s trichrome staining to assess the degree of atrial interstitial fibrosis. Atrial fibrosis was assessed as described in a previous study (Qiu et al., 2018b). The total left ventricular fibrosis area (%) in the rats from each group was calculated using the Image-Pro Plus version 6.0 software (Media Cybernetics, Silver Spring, MD, USA).

### Real-time reverse transcription-polymerase chain reaction

Total RNA from heart tissues were extracted using TRIzol reagent (Invitrogen, Carlsbad, CA, USA) according to the manufacturer’s instructions. Total RNA of heart tissues were converted into cDNA using a commercial cDNA reverse transcription kit (cat.RR037A; TAKARA Bio Inc., Otsu, Shiga, Japan), and then were detected using a SYBR Green kit (cat.RR420A;TAKARA Bio Inc., Otsu, Shiga, Japan) on ABI PRISM 7900 machine (Applied Biosystems, Foster City, CA, USA), as described previously (Ding et al., 2018). The cycle threshold (CT) values were determined in triplicates and the 2^−△△CT^ method was used to calculate the gene expression levels. Sequences of real-time PCR primers were presented in Table 1.

**Table 1:** Sequences of real-time PCR primers.

Gene	Forward primer (5′–3′)	Reverse primer (5′–3′)
*NLRP3*	GAGCTGGACCTCAGTGACAATGC	ACCAATGCGAGATCCTGACAACAC
*ASC*	AGAGTCTGGAGCTGTGGCTACTG	ATGAGTGCTTGCCTGTGTTGGTC
*Caspase-1*	ATGGCCGACAAGGTCCTGAGG	GTGACATGATCGCACAGGTCTCG
*TGF*-*β1*	CAGAGAAGAACTGCTGTGTACGG	CAGACAGAAGTTGGCATGGTAGC
*SMAD2*	CCAGGTCTCTTGATGGTCGT	TGATAAACGGCCTCAAAACC
*β*-*actin*	TCCCTGTATGCCTCTGGTCG	GTGGTGGTGAAGCTGTAGCC

**Notes.**

*NLRP3* the nucleotide-binding domain and leucine-rich repeat protein 3 ASC apoptosis-associated speck-like protein containing a caspase activation *recruitment domain* TGF-*β*1 transforming growth factor-*β*1

### Western blotting analysis

The heart tissues were homogenized with RIPA protein extraction reagent (Beyotime, Jiangsu, China). Equal quantities of the heart protein samples were separated via sodium dodecyl sulfate polyacrylamide gel electrophoresis (SDS-PAGE) on 10 or 12% polyacrylamide gels; the resultant protein bands were transferred onto immobilon-P polyvinylidene difluoride (PVDF) membranes. The membranes were blocked with 5% nonfat milk for 2 h, and were then probed with the following primary antibodies: anti-NLRP3 (diluted 1:1,000), anti-ASC (diluted 1:1,000), anti-caspase-1 p10 (diluted 1:1,000), anti-TGF-*β*1(diluted 1:1,000), anti-SMAD-2 (diluted 1:1,000), anti-p-SMAD-2 (Ser465/467) (diluted 1:1,000), and anti-GAPDH antibodies (diluted 1:10,000) overnight at 4 °C. The membranes were then washed thrice for 10 min and incubated with the corresponding secondary antibodies. The protein bands were visualized by using enhanced chemiluminescence detection kits. The results of the western blotting analyses were quantified using ChemiDoc Quantity One software (Bio-Rad Laboratories, Milan, Italy). *β*-actin served as the loading control.

### Statistical analysis

SPSS13.0 (SPSS, Chicago, IL, USA) was used to analyze the results. All values are expressed as the means ±  standard deviations (SDs). One-way analysis of variance (ANOVA), followed by post hoc analysis (Bonferroni pos *t*-test), was used to analyze the differences between the data from the groups. Statistical significance was defined at *P* < 0.05.

## Results

### RES reduced the BNP, IL-6, CRP, and TNF-*α* levels in the serum

**Figure 1:**
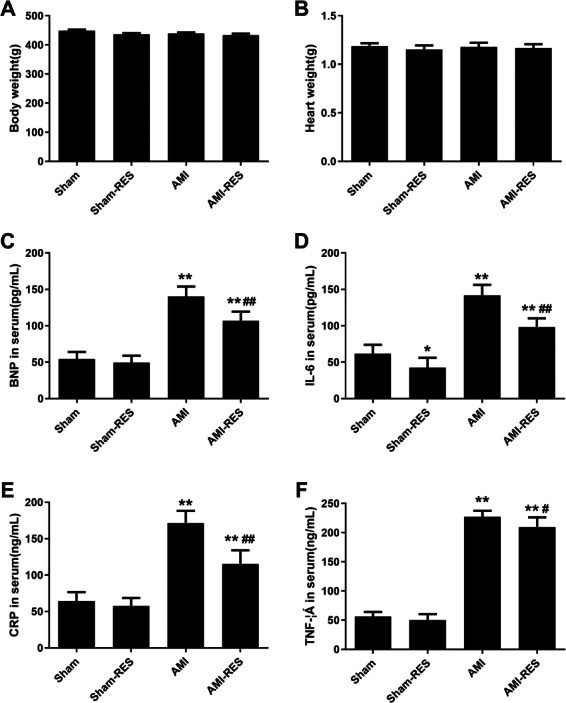
The serum levels of BNP, IL-6, CRP, and TNF-*α* (*n* = 8–10 samples per group). (A) Serum BNP levels. (B) Serum IL-6 levels. (C) Serum CRP levels. (D) Serum TNF-*α* levels. The data are expressed as the means ±  SDs. ^∗∗^, *P* < 0.01 compared with the Sham group.^#^, *P* < 0.05 and ^##^, *P* < 0.01 compared with the AMI group.

The levels of serum biomarkers were determined at 45 days post the induction of AMI. Compared to the rats from the Sham group, the serum levels of biomarkers including BNP, IL-6, CRP, and TNF- *α* increased significantly (*P* <  0.01) in the rats with AMI (Fig. 1). Moreover, the serum levels of BNP, IL-6, CRP, and TNF-*α* in the rats from the AMI-RES group decreased significantly, compared to the case for those from the AMI group (*P* <  0.01 or *P* <  0.05) (Fig. 1). In contrast, RES only decreased the serum IL-6 levels in rats from the Sham group (*P* <  0.01) (Fig. 1B). No significant difference between the serum BNP, CRP, and TNF-*α* levels in rats from the Sham and Sham-RES groups was observed (*P* >  0.05).

### RES improved the cardiac function of rats with AMI

**Figure 2:**
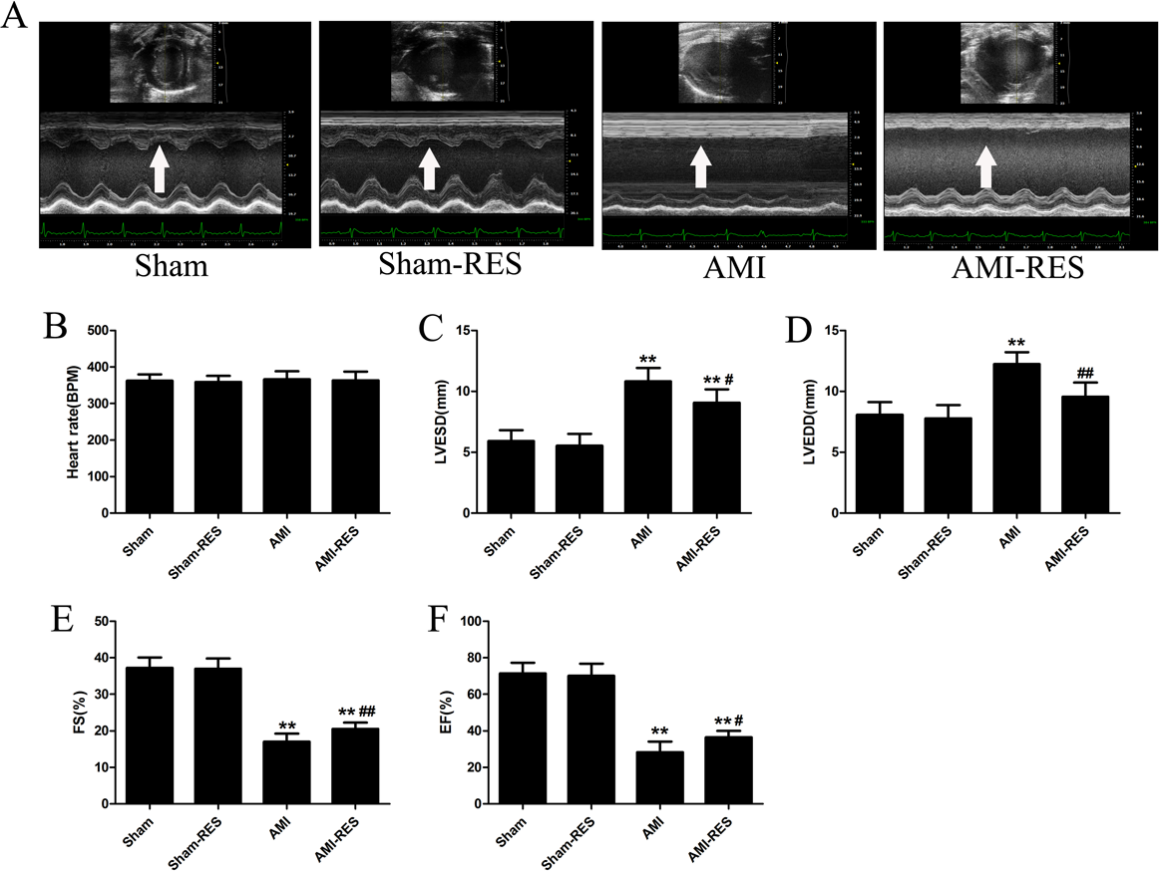
Echocardiographic parameters (*n* = 5 rats per group). (A) Typical echocardiographic images of the hearts of rats from the Sham, Sham-RES, AMI, and AMI-RES groups. (B) Heart rate (HR). (C) Left ventricular end-systolic diameter (LVESD). (D) Left ventricular end-diastolic diameter (LVEDD). (E) Fractional shortening (FS). (F) Ejection fraction (EF). The data are expressed as the means ± SDs. ^∗^, *P* < 0.05 and ^∗∗^, *P* < 0.01 compared with the Sham group. ^#^, *P* < 0.05 and ^##^, *P* < 0.01 compared with the AMI group.

We used echocardiography to assess the protective effects of RES treatment following AMI using various cardiac function parameters. As shown in Fig. 2B, there was no difference between the heart rates of the rats from the four groups (*P* >  0.05). In case of the rats from the AMI group, the LVESD and LVEDD increased significantly (*P* <  0.01) (Figs. 2C–2D), while the FS and EF decreased significantly (*P* <  0.01) (Figs. 2E–2F). Furthermore, RES treatment markedly reduced the LVESD and LVEDD, and significantly increased the FS and EF of the rats (*P* <  0.01 or *P* <  0.05) (Figs. 2C–2F). However, no significant difference between the LVESD, LVEDD, FS, and EF of rats from the Sham and Sham-RES groups was observed (*P* >  0.05).

### RES suppressed ventricular fibrosis in rats with AMI

**Figure 3:**
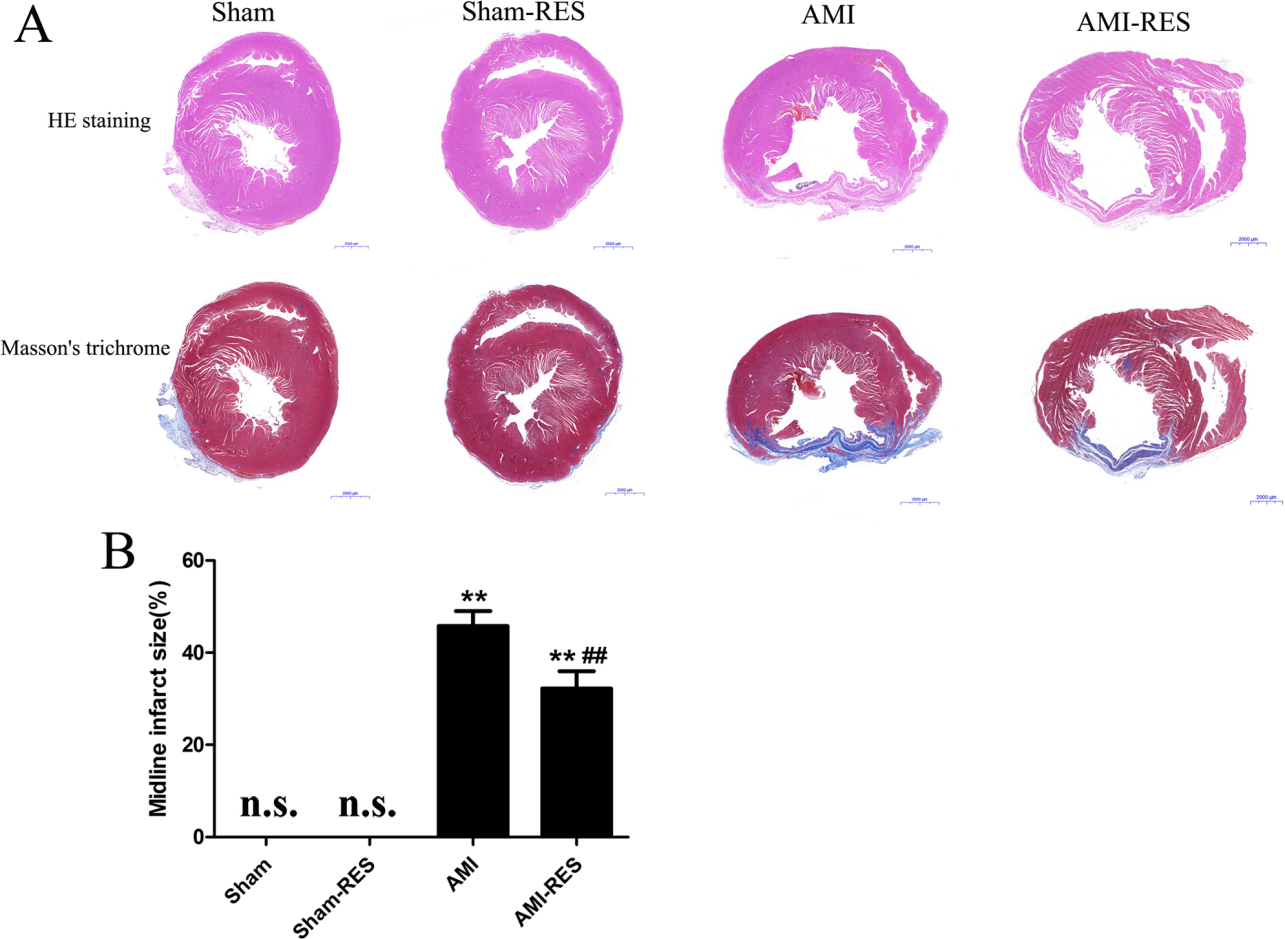
Assessment of atrial fibrosis in the rats from the four groups. (A) H&E staining (scale bar = 50 µm). (B) Masson’s trichrome staining of collagen fibers (scale bar = 50 µm). (C) Quantification of atrial fibrosis (*n* = 3 samples per group). The data are expressed as the means ±  SDs. ^∗∗^, *P* < 0.01 compared with the Sham group. ^##^, *P* < 0.01 compared with the AMI group. n.d., not detectable.

As shown in Fig. 3, we observed the histology of the heart tissues of the rats by H&E staining and evaluated the degree of left ventricular fibrosis in the rats by Masson’s trichrome staining. The degree of left ventricular fibrosis in the rats from the AMI group was significantly higher than that in the rats from the Sham group (*P* <  0.01) (Fig. 3B). Compared to the case for the rats from the Sham group, treatment with RES significantly reduced the degree of left ventricular fibrosis in the rats with AMI (*P* <  0.01) (Fig. 3B).

### RES inhibited NLRP3 inflammasome activation in the heart tissues

**Figure 4:**
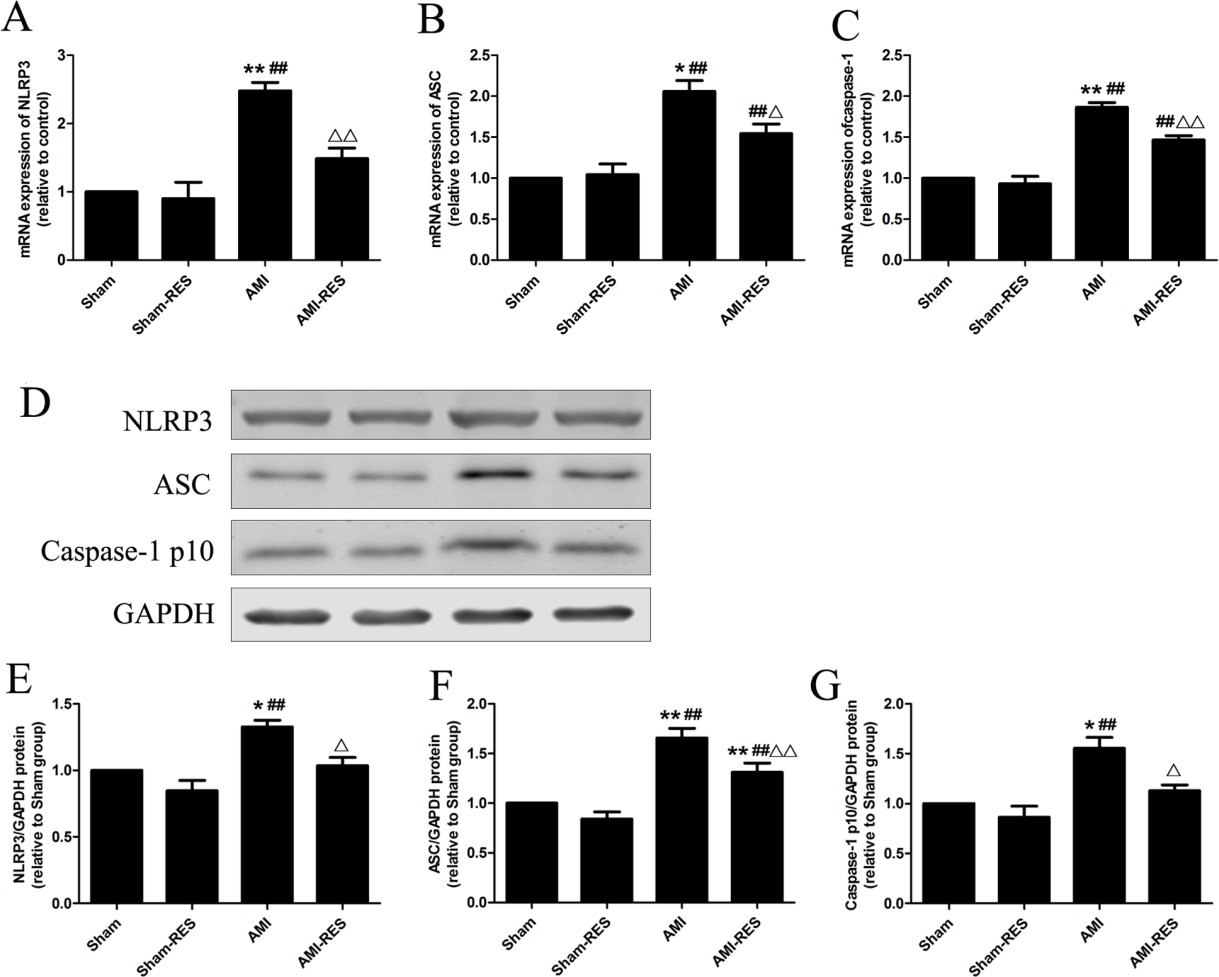
RES inhibits NLRP3 inflammasome activation in the heart tissues of rats with AMI (*n* = 3 samples per group). (A) The mRNA expression of NLRP3. (B) The mRNA expression of ASC. (C) The mRNA expression of caspase-1. (D) Representative images of the western blotting analysis for the quantification of NLRP3, ASC, and caspase-1 p10. (E) The protein expression of NLRP3. (F) The protein expression of ASC. (G) The protein expression of caspase-1 p10. The data are expressed as the means ±SDs. ^∗^, *P* < 0.05 and ^∗∗^, *P* < 0.01 compared with the Sham group. ^#^, *P* < 0.05 and ^##^, *P* < 0.01 compared with the AMI group. ^△^, *P* < 0.05 and ^△△^, *P* < 0.01 compared with the AMI group.

To assess the NLRP3 inflammasome activity, we determined the protein expression levels of NLRP3, ASC, and caspase-1 p10 in heart tissues. Refer to Fig. 4, compared to the rats from the Sham group, the protein expressions of NLRP3, ASC, and caspase-1 p10 in heart tissues were markedly increased in the rats with AMI (*P* <  0.05 or *P* <  0.01) (Fig. 4). Compared to the AMI group, RES supplementation significantly decreased the protein expressions of NLRP3, ASC, and caspase-1 p10 in the heart tissues of AMI-RES group (*P* <  0.05 or *P* <  0.01). Furthermore, these above protein expressions showed no significant difference between the Sham group and the Sham-RES group (*P* >  0.05).

### RES inhibited the TGF-*β*1/Smad2 pathway in the heart tissues

**Figure 5:**
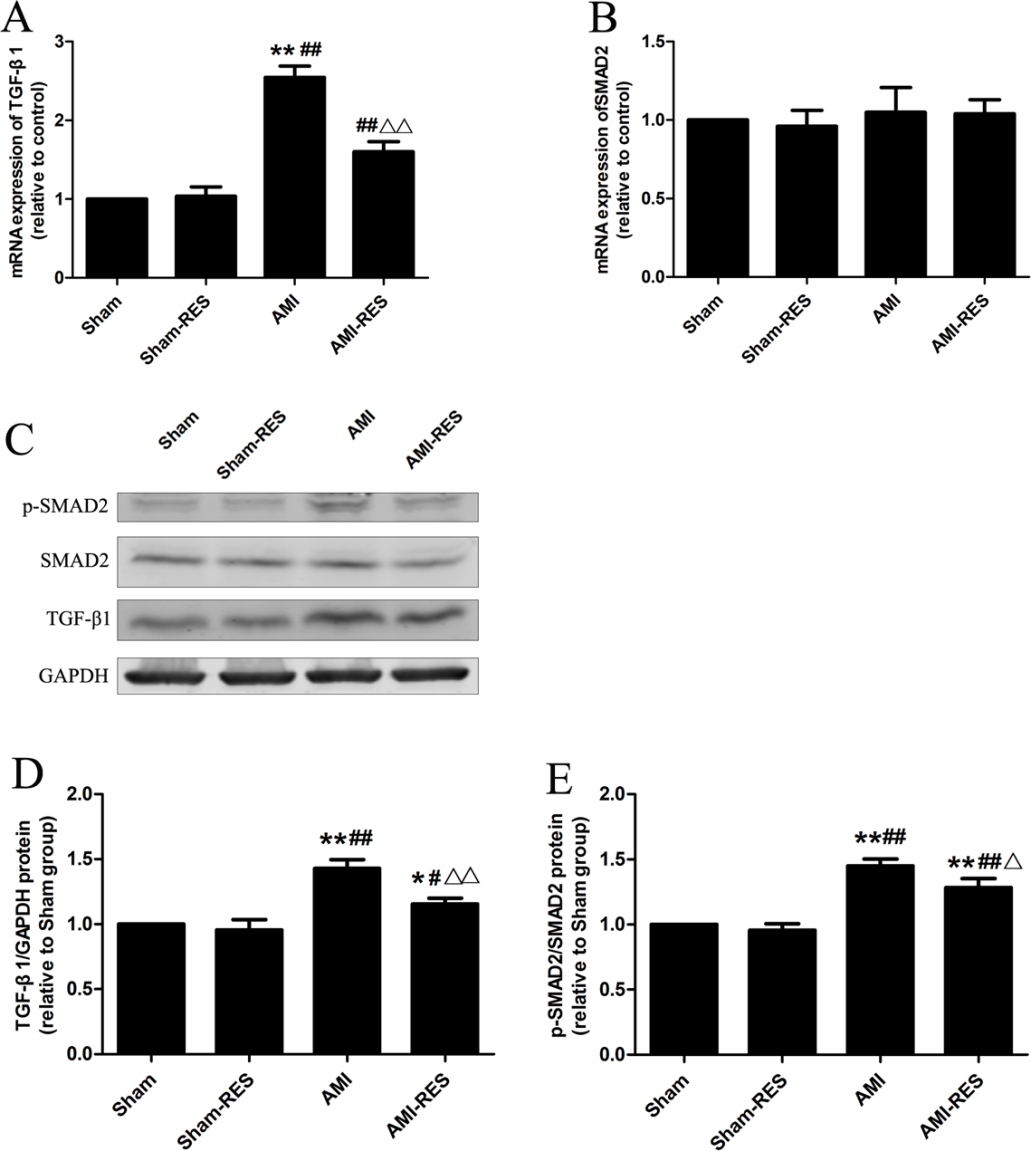
RES inhibits the TGF-*β*.1/SMAD2 signaling pathway-mediated fibrosis (*n* = 3 samples per group). (A) The mRNA expression of TGF-*β*1. (B) The mRNA expression of SMAD2. (C) Representative images of the western blotting analysis for the quantification of TGF-*β*1, *p*-SMAD2, and SMAD2 in the heart tissues. (D) The protein expression of TGF-*β*1 in the heart tissues. (E) The protein expression of *p*-SMAD2/SMAD2 in the heart tissues. The data are expressed as the means ±  SDs. ^∗^, *P* < 0.05 and ^∗∗^, *P* < 0.01 compared with the Sham group. ^#^, *P* < 0.05 and ^##^, *P* < 0.01 compared with the AMI group. ^△^, *P* < 0.05 and ^△△^, *P* < 0.01 compared with the AMI group.

Figure 5 shows that the protein expressions of TGF-*β*1 and p-SMAD2 in heart tissues of rats in the AMI group were markedly increased than that in the Sham group (*P* <  0.01). Moreover, the heart tissues of rats in the AMI-RES exhibited markedly lower protein expressions of TGF-*β*1 and p-SMAD2 than the AMI group (*P* <  0.05 or *P* <  0.01). No significant difference of the protein expressions of TGF-*β*1 and p-SMAD2 in heart tissues were observed between the Sham group and the Sham-RES group (*P* >  0.05).

## Discussion

The main finding of our study is that pretreatment and treatment with a moderate-high dose of RES (50 mg/kg/day) during the post-infarction period partially reversed the adverse left ventricular remodeling and improved the cardiac performance observed following a large myocardial infarction (MI) in rats. Our results suggest that these beneficial effects of RES are mainly attributed to its ability to inhibit NLRP3 inflammasome activation, as well as suppress the TGF-*β*1/SMAD2 signaling pathway.

AMI originally caused injury by reducing the blood supply to the tissues, and then further triggered an extensive inflammatory response that occurs during organ reperfusion. Unfortunately, excessive left ventricular remodeling in the heart during the post-infarction period has been shown to cause the loss of cardiac function and heart failure (Pfeffer, 1995). Cardiac dysfunction caused by AMI is the leading cause of the morbidity and mortality associated with CVDs worldwide (Velagaleti et al., 2008). Additionally, AMI-induced acute and chronic cardiac remodeling will result in structural and functional abnormalities such as left ventricular dilatation and systolic dysfunction (French & Kramer, 2007). Thus, reversing ventricular remodeling is an attractive strategy to prevent adverse clinical outcomes of AMI and ensure favorable long-term prognoses in AMI patients. The process of ventricular remodeling is complex; fibrosis plays an important role in ventricular remodeling and heart failure (Passino et al., 2015). It is well known that RES exerts multiple health-beneficial effects by regulating a variety of key molecules (Bonnefont-Rousselot, 2016; Zordoky, Robertson & Dyck, 2015). Some in vivo studies on rodent and swine models of ischemia/reperfusion have reported the beneficial effects of RES treatment against ischemic heart disease (Raj et al., 2014). A previous study has reported that treatments with low doses of RES (2.5 mg/kg/day) or perindopril (2.5 mg/kg/day) for 8 weeks were equally effective in significantly alleviating adverse cardiac remodeling and improving contractile dysfunction in rats with AMI (Raj et al., 2016). Moreover, RES (50 mg/kg/day) has been shown to partially reverse MI-induced remodeling (left ventricular dilation) in hearts by enhancing the autophagy-activating AMP-kinase pathway and significantly improve cardiac function (Kanamori et al., 2013). These above studies provide a strong rationale to hypothesize that RES treatment may ameliorate the progression of adverse cardiac remodeling following MI. Consistent with these findings, we also found that the administration of a high dose of RES (50 mg/kg/day) daily for 7 days before AMI induction and for 45 days following AMI significantly improved the echocardiographic parameters including LVESD, LVEDD, EF, and FS, and alleviated left ventricular fibrosis in a rat model of AMI. Although Burstein et al. (2007). have found that RES (17 mg/kg/day) treatment for 12 weeks failed to reduce the infarct size and improve the deterioration of hemodynamic function or echocardiographic indices of cardiac remodeling following MI, the preservation of the contractile reserve observed in response to dobutamine stress suggests that RES may exert protective effects against cardiac remodeling following MI. These inconsistent results regarding the effects of RES treatment on cardiac remodeling following MI may be due to the differences in the RES dose administered and/or the intervention procedures with RES.

Recently, an increasing number of evidence showed that NLRP3 inflammasome is associated with many diseases, including cardiovascular diseases, inflammatory issues (such as liver diseases and inflammatory bowel diseases), neurologic disorders, endometriosis and pseudomonas aeruginosa infection (Fusco et al., 2018; Fusco et al., 2020; Gugliandolo et al., 2019). The NLRP3 inflammasome is a macromolecular protein complex; it belongs to the inflammasome family. It has been demonstrated that NLRP3 inflammasome mediates caspase 1 activation and the production and secretion of IL-1*β* and IL-18 (powerful pro-inflammatory cytokines) (Toldo et al., 2015), which forms a positive feedback loop to promote TGF-*β*1 activation (Fix, Bingham & Carver, 2011; Qiu et al., 2018a). In addition, TGF-*β*1 has been established as a key profibrotic factor involved in post-MI remodeling and is accessible via the regulation of the phosphorylation of SMADs (Gramley et al., 2010; Sui, Wei & Wang, 2015). This evidence indicates that the NLRP3 inflammasome may have profibrogenic effects, which may regulate the production and maturation of IL-1*β* and IL-18/TGF-*β*1-SMADs. As an integral part of the innate immune system, the NLRP3 inflammasome is involved in AMI progression and the typical response to AMI-induced injury (Bullon et al., 2017; Toldo & Abbate, 2018). Matsumura et al. (2018) have reported that MI induced left ventricular and atrial remodeling in a rat model of AMI, and that RES treatment significantly ameliorated the AMI-induced cardiac remodeling and heart failure. Consistent with previous findings, we found that MI induced left ventricular fibrosis, and that RES supplementation significantly reduced the MI-induced left ventricular remodeling. We further found that following AMI, the gene and protein expression levels of NLRP3, ASC, caspase-1, and TGF-*β*1, and the phosphorylation of SMAD2 increased markedly, suggesting that the activation of the NLRP3 inflammasome and TGF-*β*1/SMAD2 signaling pathway was associated with left ventricular remodeling in a rat model of AMI at 45 days after MI. Several previous studies have reported that the inhibition of NLRP3 inflammasome activity shows beneficial effects in rodent models of AMI (Mezzaroma et al., 2011; Toldo et al., 2016). In this study, RES treatment significantly reduced the activation of the NLRP3 inflammasome and decreased the expression of TGF-*β*1 and the phosphorylation of SMAD2, suggesting that the antifibrotic mechanisms of RES may involve the inhibition of NLRP3 inflammasome activation and suppression of the TGF-*β*1/SMAD2 signaling pathway. It has been reported that hyperglycemia exacerbates ipilimumab-caused cardiotoxicity and reduces its anticancer efficacy in breast cancer cells (MCF-7 and MDA-MB-231 cells) in a NLRP3-sensitive manner (Ahmet et al., 2017). Notably, NLRP3 inflammasome may be as potential target to prevent cardiopulmonary complications in patients with SARS-CoV-2 (an enveloped and non-segmented RNA based virus) (Quagliariello et al., 2020). Thus, RES can inhibit the NLRP3 inflammasome activation, which makes it potential application for decreasing cardiac injury in breast cancer patients and in management of patients with SARS-CoV-2 infection.

In myocardial fibroblasts following infarction, NLRP3 inflammasome-related proteins are up-regulated, which may contribute to infarct size in ischemia-reperfusion injury (Sandanger et al., 2013). Thus, NLRP3 inflammasome-mediated inflammation may play vital role in ischemia-reperfusion injury. Inflammation has been demonstrated as the major factor involved in the pathophysiology of the MI-induced injury cascade, including cardiac remodeling and dysfunction (Liu, Wang & Li, 2016). It has been reported that the levels of IL-6, CRP, and TNF-*α* are important indicators of MI-related cardiac complications, and these inflammatory cytokines have been reported to be closely associated with the occurrence of heart failure (Chen et al., 2011; Danesh et al., 2004; Groot et al., 2015). RES has been demonstrated to significantly decrease the secretion of proinflammatory cytokines through multiple signal pathways in cardiac tissue (Berretta et al., 2020). In addition, some previous experiments have reported that RES treatment improved the proinflammatory status by reducing the TNF-*α* level in hypertensive rats (Thandapilly et al., 2013) and rats with MI (Raj et al., 2016). Consistent with the results of previous studies, we found that RES treatment markedly decreased the levels of IL-6, CRP, and TNF-*α* in a rat model of AMI.

## Conclusions

In summary, RES treatment significantly improves the cardiac function and left ventricular fibrosis in rats with AMI; the mechanisms underlying this phenomenon may involve the inhibition of NLRP3 inflammasome activation and suppression of the TGF- *β*1/SMAD2 signaling pathway. These findings may constitute a novel upstream therapy for AMI, which involves the suppression of left ventricular fibrosis.

##  Supplemental Information

10.7717/peerj.11501/supp-1 Supplemental Information 1 Raw data

10.7717/peerj.11501/supp-2 Supplemental Information 2 Raw data 2

10.7717/peerj.11501/supp-3 Supplemental Information 3 Author checklist

## Additional Information and Declarations

### Data Availability

The following information was supplied regarding data availability: The raw measurements are available in the Supplementary Files.
