# Supplementary figures and images for: Resveratrol improves cardiac function and left ventricular fibrosis after myocardial infarction in rats by inhibiting NLRP3 inflammasome activity and the TGF-β1/SMAD2 signaling pathway
Source: PeerJ. 2021 May 28;9:e11501. doi: 10.7717/peerj.11501 (PMC8166236; doi:10.7717/peerj.11501)

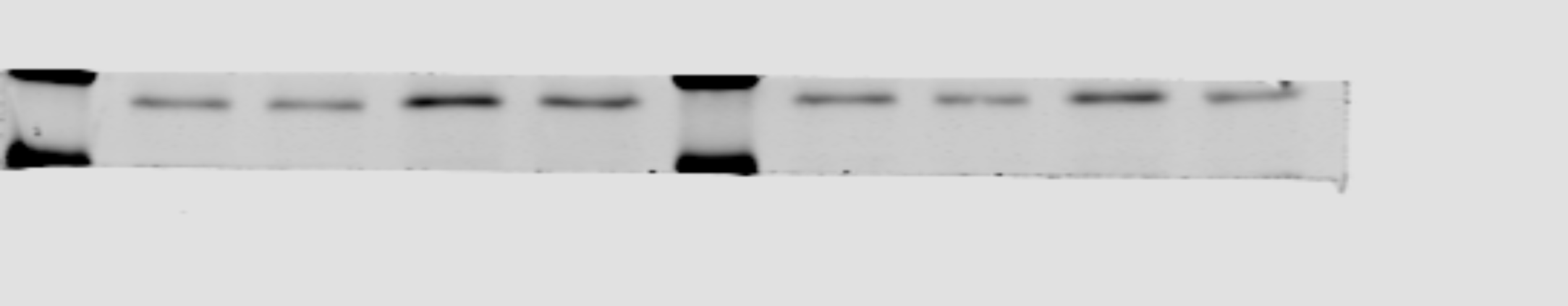

Supplement: Supplemental Information 2 [file peerj-09-11501-s002.zip › Supplementary File 2/ASC-2.tif.tif]

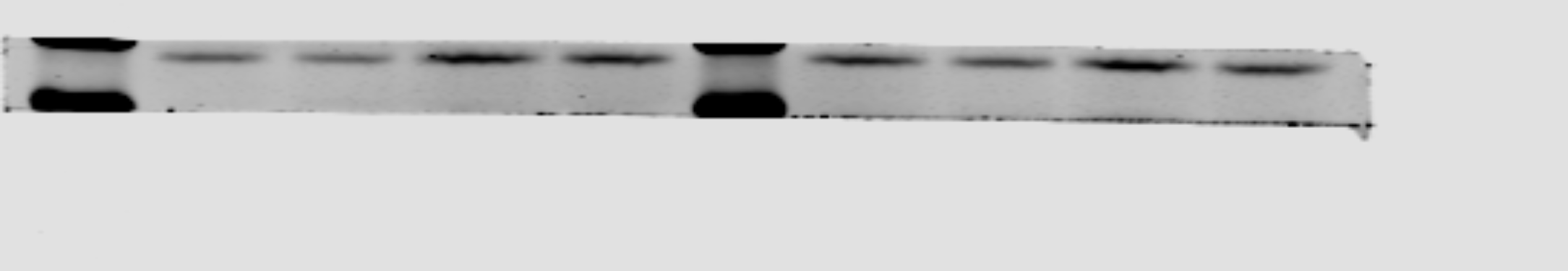

Supplement: Supplemental Information 2 [file peerj-09-11501-s002.zip › Supplementary File 2/ASC.tif.tif]

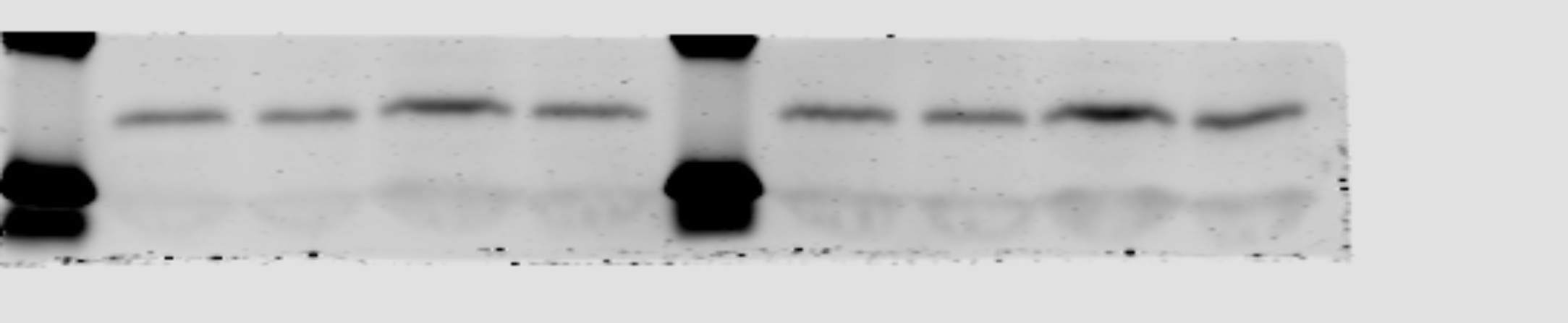

Supplement: Supplemental Information 2 [file peerj-09-11501-s002.zip › Supplementary File 2/Caspase1 p10-2.tif.tif]

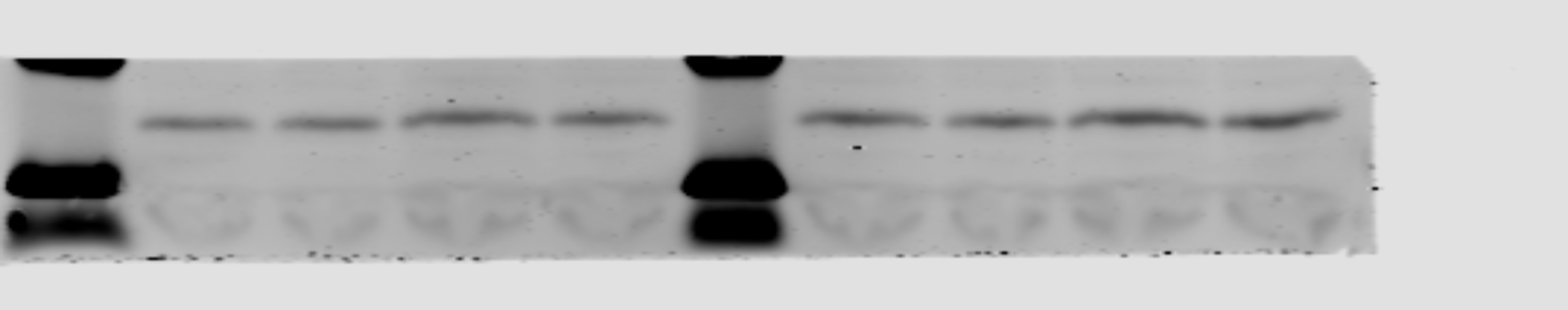

Supplement: Supplemental Information 2 [file peerj-09-11501-s002.zip › Supplementary File 2/Caspase1 p10.tif.tif]

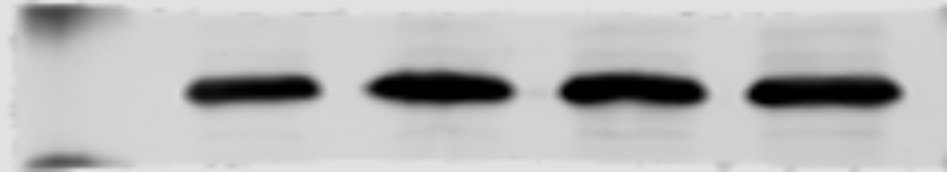

Supplement: Supplemental Information 2 [file peerj-09-11501-s002.zip › Supplementary File 2/GAP-1.png]

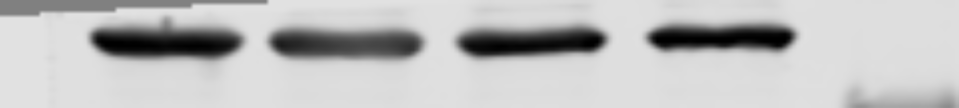

Supplement: Supplemental Information 2 [file peerj-09-11501-s002.zip › Supplementary File 2/GAP-2.png]

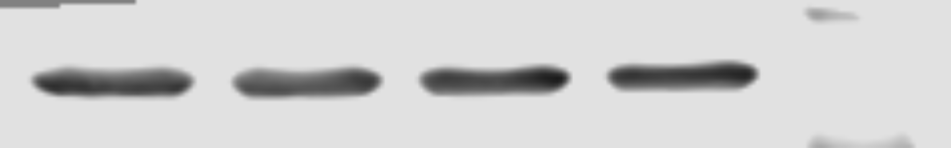

Supplement: Supplemental Information 2 [file peerj-09-11501-s002.zip › Supplementary File 2/GAP-3.png]

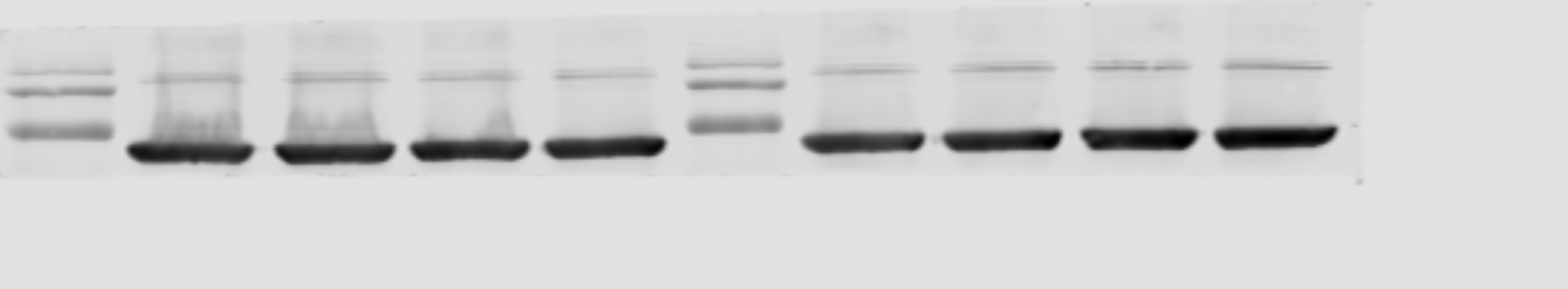

Supplement: Supplemental Information 2 [file peerj-09-11501-s002.zip › Supplementary File 2/GAPDH-1.tif.tif]

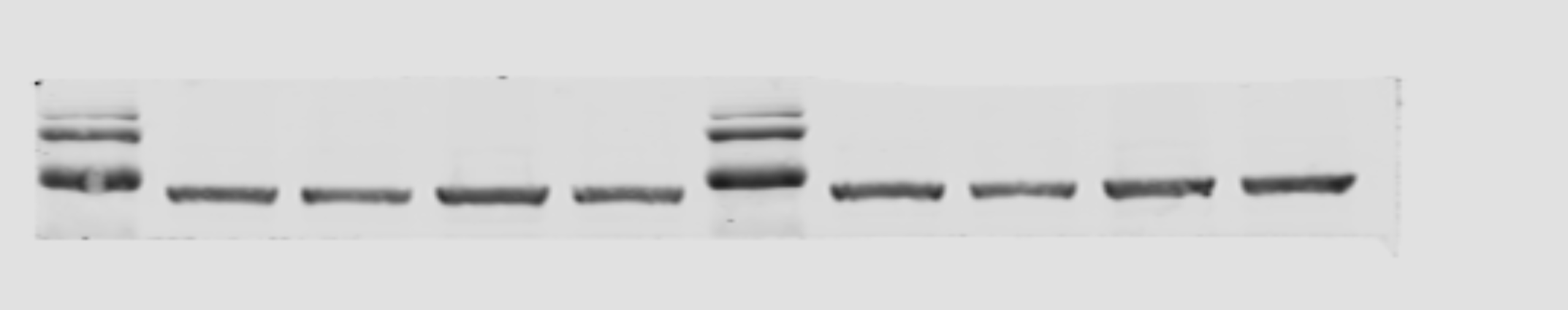

Supplement: Supplemental Information 2 [file peerj-09-11501-s002.zip › Supplementary File 2/NLRP3-2.tif.tif]

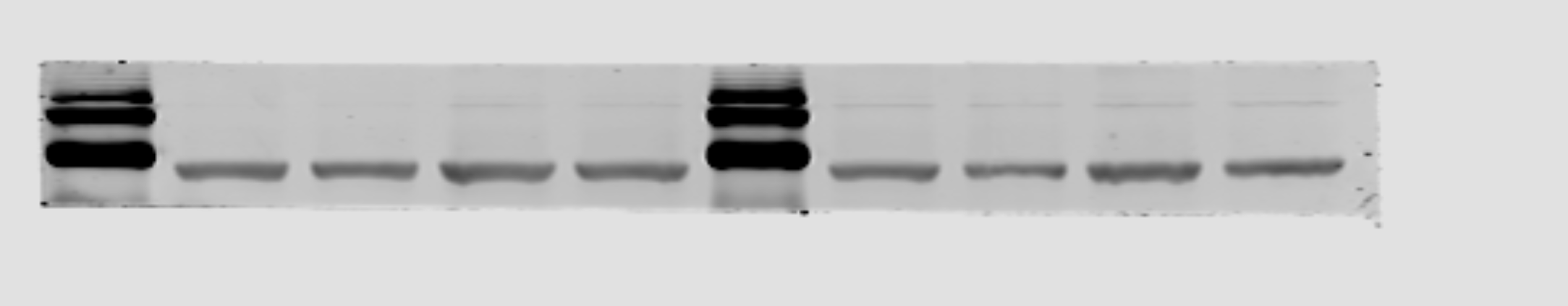

Supplement: Supplemental Information 2 [file peerj-09-11501-s002.zip › Supplementary File 2/NLRP3.tif.tif]

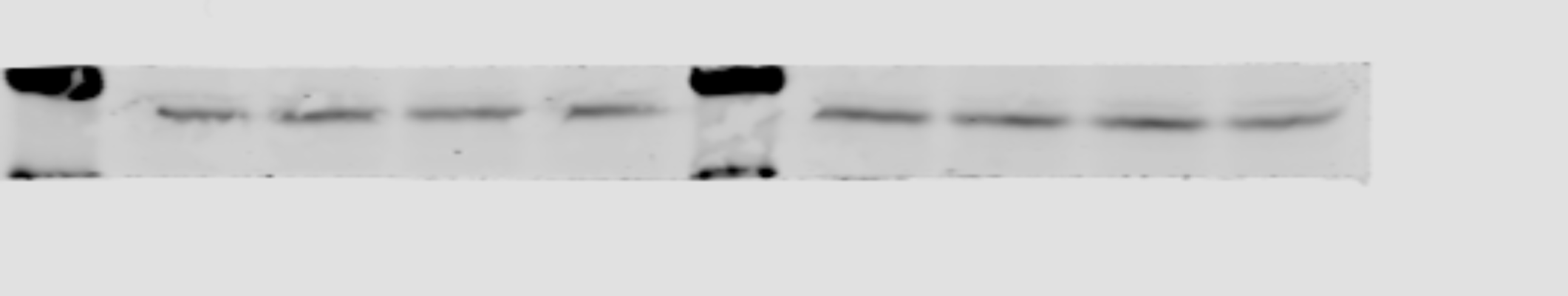

Supplement: Supplemental Information 2 [file peerj-09-11501-s002.zip › Supplementary File 2/SMAD2-1.tif.tif]

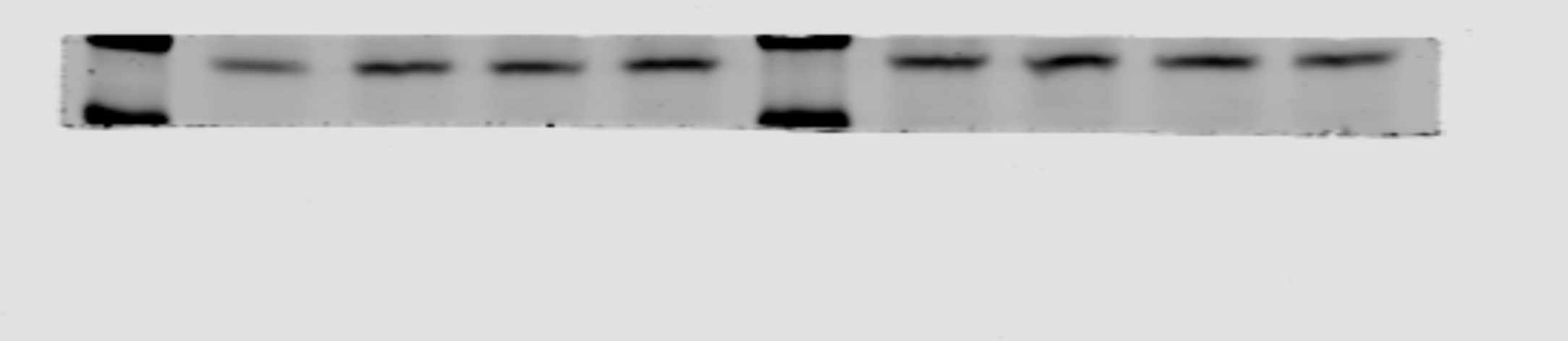

Supplement: Supplemental Information 2 [file peerj-09-11501-s002.zip › Supplementary File 2/SMAD2-2.tif.tif]

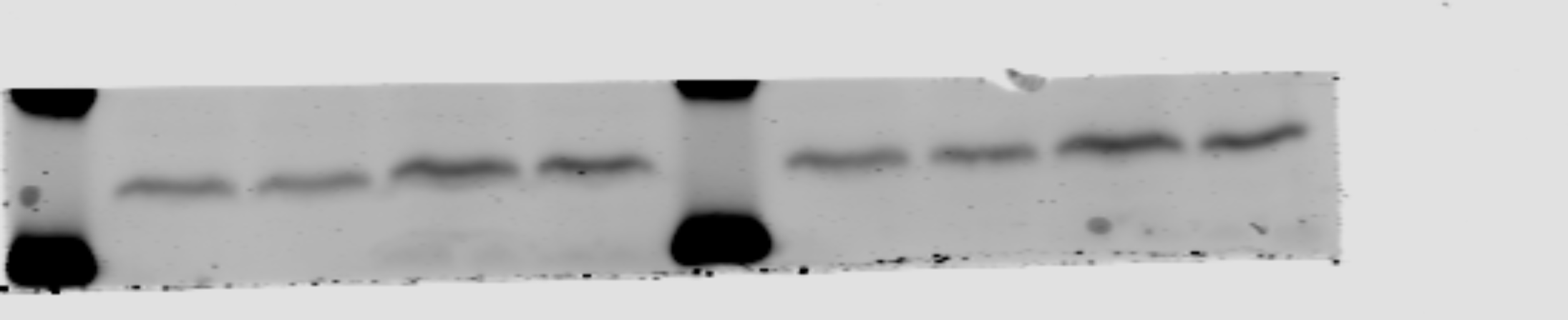

Supplement: Supplemental Information 2 [file peerj-09-11501-s002.zip › Supplementary File 2/TGF-beta-1.tif.tif]

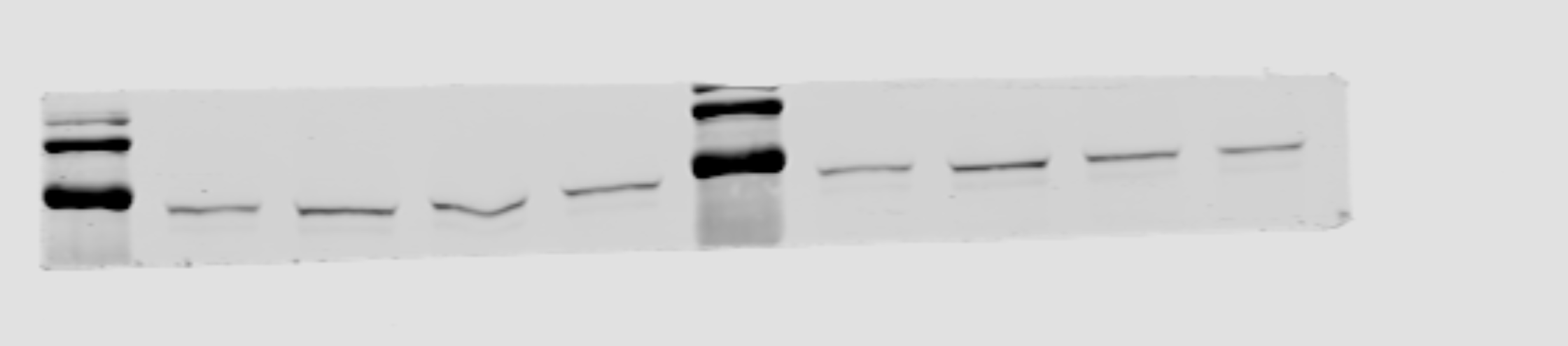

Supplement: Supplemental Information 2 [file peerj-09-11501-s002.zip › Supplementary File 2/TGF-beta-2.png]

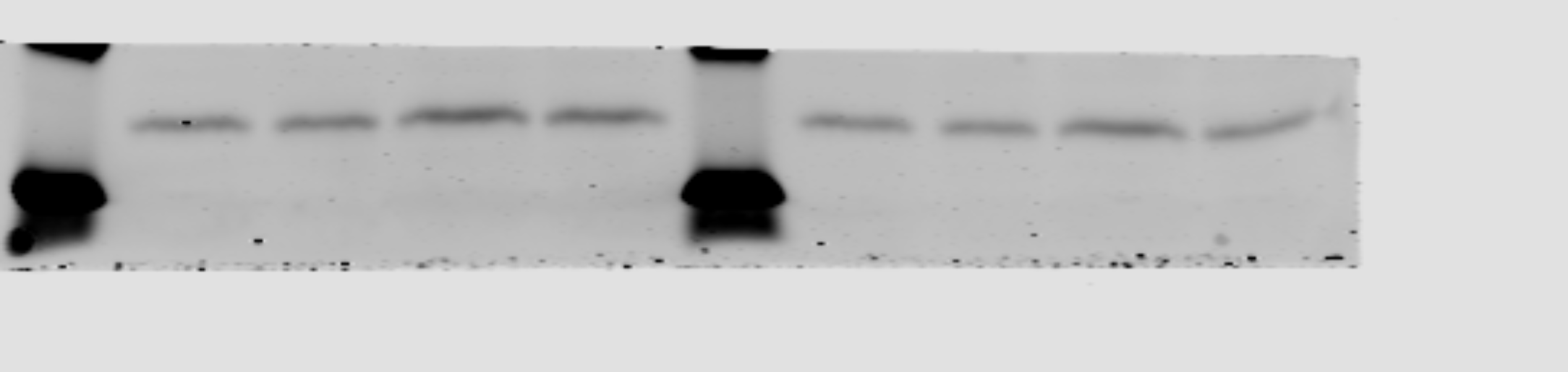

Supplement: Supplemental Information 2 [file peerj-09-11501-s002.zip › Supplementary File 2/pSMAD2-2.tif.tif]

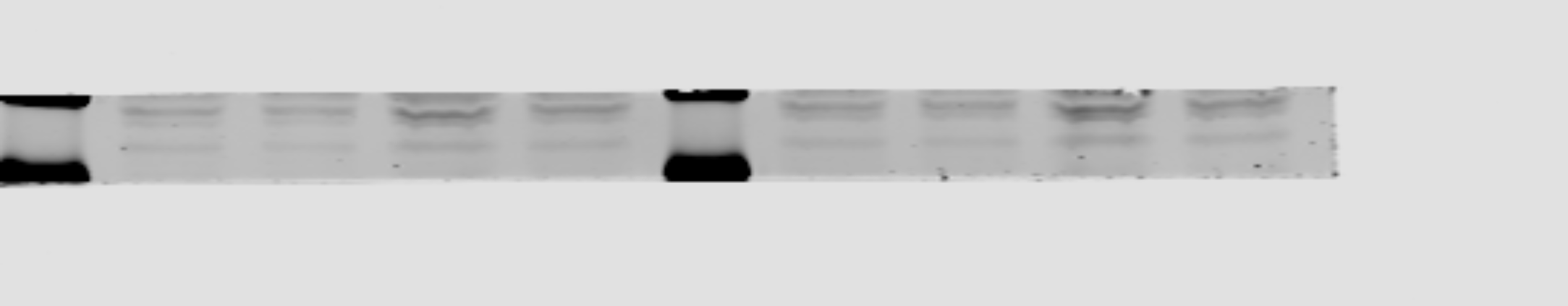

Supplement: Supplemental Information 2 [file peerj-09-11501-s002.zip › Supplementary File 2/pSMAD2.tif.tif]
